# Stay Home: Role of Physical Exercise Training in Elderly Individuals' Ability to Face the COVID-19 Infection

**DOI:** 10.1155/2020/8375096

**Published:** 2020-11-28

**Authors:** Walid Kamal Abdelbasset

**Affiliations:** ^1^Department of Health and Rehabilitation Sciences, College of Applied Medical Sciences, Prince Sattam bin Abdulaziz University, Al-Kharj, Saudi Arabia; ^2^Department of Physical Therapy, Kasr Al-Ainy Hospital, Cairo University, Giza, Egypt

## Abstract

Recently, the novel coronavirus epidemic occurred in China and spread worldwide to become a global pandemic. COVID-19 is a fatal viral infection causing death, particularly in aged individuals, due to impaired immunity. To date, no intervention is available to prevent COVID-19 and its manifestations. Physical exercise training generally has health benefits, and it assists in the prevention of several chronic diseases. Therefore, this review is aimed at exploring the role of physical exercise training in the face of COVID-19 in older adults and elderly individuals. From this point of view, this review suggests that physical exercise training plays a key role in promoting immune system regulation, delaying immunity dysfunction, reducing circulatory inflammation markers, and preventing sarcopenia and thus could prevent the risk of acquiring COVID-19 infection and reduce the complications of recommended self-isolation in older adults and elderly individuals. Additionally, immunity biomarkers were optimistically demonstrated in older adults following physical exercise training, thereby reducing mortality and morbidity rates. Finally, in accordance with recommendations to stay home and perform self-isolation to prevent the spread of COVID-19, all populations are strongly recommended to practice regular home exercise training at home to promote immune system functioning.

## 1. Introduction

Recently, in December 2019, the novel coronavirus disease (COVID-19) occurred in China [1] and subsequently spread worldwide to become a global pandemic [2]. Generally, COVID-19 is identified as an acute disorder that could be fatal. The onset of this severe disease may lead to death because of the substantial damage to lung alveoli and the massive failure of the respiratory system to conduct its functional gas exchange [3].

As of April 11, approximately 1746607 individuals have been confirmed to have COVID-19, and approximately 107327 deaths have been reported globally [2]. Therefore, most countries worldwide have recommended individuals to stay home and practice self-isolation to control and avoid the spread of COVID-19 infection [4]. However, these recommendations may result in a lack of physical activity which may lead to physical and psychological complications [5]. Self-isolation may lead to an increase in anxiety and depression rates [6], particularly among older adults [7] who are most affected by this pandemic. In addition, the lack of physical activity may lead to increased blood glucose levels, vulnerability to infection, cardiovascular disorders, cognitive dysfunction, and musculoskeletal disease [8]. Fighting the COVID-19 pandemic may continue for a prolonged time, and this event may worsen the psychological and mental status including anger, hostility, hopelessness, and depression that can lead to cardiovascular or cerebrovascular dysfunction and intensify mortality rate [9]. Moreover, aging is associated with loss of muscle mass, decreased muscle strength, and impaired functional activity known as sarcopenia [10], which may be increased with this isolation, increase mortality, and reduce quality of life due to self-isolation and lack physical activity [11, 12]. In this situation, while it is extremely difficult to conduct exercise interventions in rehabilitation centers, physical exercise is the main modality to overcome the complications of the recommended self-isolation [13].

While the pathological mechanism of COVID-19 is not completely understood, virus and host factors play a critical role in COVID-19 infection. During a viral infection, the immune system becomes active to fight the virus and its predisposing factors. However, immunopathogenesis is commonly related to the control of the immune response, which leads to damage to lung tissues, in turn disturbing pulmonary function and impairing lung volume. A misdirected or inefficient immunity heightens viral reproduction and results in further damage to lung tissues. Furthermore, an overactive immune response may also cause immunological disorder [14]. Therefore, this review is aimed at clarifying the role of physical exercise training in the face of COVID-19 infection.

## 2. Immunity in Aging

Over the last few years, life expectancy rates have increased significantly worldwide, resulting in a high incidence of age-related infectious diseases. COVID-19 has a high fatality among older adult individuals, highlighting the potential role of preserving the integrative function of the immune system in this population [15]. Resistance to viral infections diminishes with aging because of the inescapable reduction or disturbance in the usual functioning of the immune system, susceptibility to infectious diseases, and impaired immune response to medications in older adults. The reasons for disturbed immune system functioning are multifactorial, indicating the appearance of permanent receptivity to importunate bacterial and viral infections, which exhaust the immune system, specifically in T- and B-cells [16].

Older adults' susceptibility to infectious diseases increases because of the considerable decline in the number of T-cells, which play an important role in identifying and reacting continuously to growing pathogens such as viral infections [17]. Although this decline is strongly associated with reduced adaptive functions of the immune system, the innate sections of the immunity cannot resist the negative influences of the aging process [18]. Additionally, inflammatory markers, including interleukins and C-reactive protein, increase with aging. Also known as inflammaging, this increase results in chronic diseases in older adults [19].

## 3. Exercise and Immunity

The present review focuses on studies that assessed the influences of conducting a regular physical exercise program on the immune system in older adults, aimed at reducing the COVID-19-related mortality rate in this population. Generally, it has been documented that regular adherence to exercise programs is related to higher life expectancy and lower risk of cardiovascular and infectious diseases [20]. Modern medicine attributes several aging-induced diseases to immune dysfunction [21]. Therefore, it would be beneficial to investigate if the effect of exercise on immunity in older adults presents significant advantages as a safe, easy, and low-cost modality to improve immunity [22]. A prior study explained that engaging in moderate-intensity aerobic exercise reduces manifestations related to upper airway infection and improves the immune system functioning system [23].

The present study is aimed at exploring the role of exercise rehabilitation in improving older adults' immunity against COVID-19. Previous studies assessed the impact of a single bout of acute exercise on different immune responses in young adults and older individuals [24, 25]. While these studies provided effective findings on older adults' immune responses to acute stress, they presented poor data on the long-term effects of physical exercise on their immunity. Other studies have examined the long-term effect of exercise intervention on the immune system in active and nonactive older adult populations [22, 26, 27]. The majority of common assessments in such studies included serum antibodies, phenotype features, number of T-cells, phagocytic function of neutrophils/monocytes, NK cell cytotoxic activity, and mitogen-induced proliferation of T-cells [28]. Another study that evaluated the influence of aerobic and resisted exercise (separated or combined) in healthy and frail middle-aged and older adults for a period of 8 to 12 weeks reported considerable and effective results for immune system response [29–31].

## 4. Exercise and Viral Infection

Viral infection provides the foremost risk for healthy and older adult survivors. Particularly, older adults are the most susceptible to severe medical complications and death as a result of COVID-19, which leads to suppression of the phagocytic activity of alveolar macrophages [2, 32]. Additionally, it leads to a decrease in the number of T-cells and reduction in the clearance by cilia, thereby increasing the severity of the infection. Improving the functional response of the immune system against COVID-19 necessitates a frequent attentive assessment because this response could already be overactive in older adults, resulting in advanced stress because of their vulnerable immunity. Although exercise training plays a vital role in preventing viral infection subsequent to the initial infection, the physiological response of the immune system is not completely known. Nevertheless, it could affect catecholamines, resulting in a change from type 1 to type 2 immune response, consequently eliminating localized inflammation and promoting good outcomes following the acquisition of viral infection [33].

Regarding the COVID-19 infection, a physical exercise program has been suggested for the period of confinement [34]. These suggestions included an increase in exercise frequency to 5-7 days of aerobic exercise and 2-3 days of resisted exercise per week. In addition, mobility, coordination, and balance exercises were recommended twice a week at least through various physical exercises. Moreover, moderate-intensity aerobic exercise was recommended for older adults during confinement without the need for particular equipment [34, 35]. Furthermore, it was suggested that yoga may be considered in this period as it does not need exercise devices or wide place [35]. Recently, it was documented that regular physical exercise training at home is a safe and important modality for promoting a healthy status, avoiding airborne COVID, and maintaining the fitness level during the staying home period [36].

## 5. Exercise and Sarcopenia

Primary sarcopenia is identified as an age-related disorder typified with a gradual loss of muscle mass, decreased muscle strength, and poor physical performance with a risk of functional disability, impaired quality of life, and increased mortality [37]. Accordingly, the mass and function of the skeletal muscles decline progressively with age. With the confinement of the COVID-19 pandemic, sarcopenia may increase due to the lack of physical activity. Poor physical performance is a potential indicator of impaired health outcomes related to aging [38]. Prior evidence approved that physical inactivity is an amendable factor for growing various chronic diseases, including obesity, diabetes, hypertension, cancer, depression, and musculoskeletal disease [39]. Exercise training is demonstrated as one of the important modalities for preventing functional impairments and chronic diseases and realizing the health status in older adults [40]. In particular, older adults with an active lifestyle and regular exercise training exhibit lower morbidity and lesser age-associated disabilities than sedentary peers [41]. Evidence suggested that exercise training could be utilized to promote physical independence in older individuals [42]. Moreover, exercise training could be prospectively used to prohibit, slow down, or reverse sarcopenia and frailty [43]. The American College of Sports Medicine suggests different exercise programs such as aerobic, resisted, balance, and coordination exercises as the ideal modalities to improve muscle function in older adults [44]. Strengthening exercise was recommended as one of the major therapeutic modalities to prevent the loss of muscle mass in aging by inducing the release of anabolic hormones that enhance protein synthesis and promote muscle strength and function [45].

## 6. Exercise and Psychosocial Impacts

The quarantine-associated COVID-19 leads to an impaired physical activity energy expenditure which harmfully impacts the psychosocial status [46]. A recent study has concluded that sedentary lifestyle may increase the susceptibility to infections; in contrast, regular home exercise training may induce preventive influences against viral infection of the respiratory airways, improve physical and mental functions, and enhance psychological condition [47]. Regarding the daily living restrictions and sedentary habits during the forced rest period of quarantine, physical activity is strongly recommended in inactive populations to improve health status and provide preventive benefits in well-being [48]. In serious issues such as the COVID-19 pandemic, physical activity practice should be the main modality for active and inactive individuals to promote and restore a healthy condition [49]. In addition, it was explained that long-term detraining may lead to significant reduction in exercise capacity and loss of muscle mass and strength [50], and thereby, athletes should avoid prolonged time of detraining to protect the health and physical condition [51]. Regular physical activity has strongly optimistic benefits on the psychological status through reducing anxiety and depression and improving stress tolerance and self-esteem [46].

## 7. Conclusions

From the author's point of view, this review suggests that physical exercise training plays a key role in promoting immune system regulation, delaying immunity dysfunction, reducing circulatory inflammation markers, and preventing sarcopenia and thus could prevent the risk of acquiring COVID-19 infection and reduce the complications of recommended self-isolation in older adults and elderly individuals. Additionally, immunity biomarkers are optimistically demonstrated in older adults following physical exercise training, thereby reducing mortality and morbidity rates in this population. Adherence to physical exercise intervention should be encouraged in the rehabilitation of patients to prevent the spread of the fatal COVID-19 infection and staying home complications, particularly among older adults. Finally, in accordance with recommendations to stay home and practice self-isolation to prevent the spread of COVID-19, all populations, and particularly older adults, are strongly recommended to conduct a regular home exercise training to promote immune system functioning. Conducting regular physical exercise is considered a main preventive modality against a quarantine complication-related COVID-19 pandemic. Therefore, “stay home stay active.”

## Data Availability

No data were used to support this study. This is a review, and no data sets were used in the manuscript.

## References

[1] Huang C., Wang Y., Li X. (2020). Clinical features of patients infected with 2019 novel coronavirus in Wuhan, China. *The Lancet*.

[2] World Health Organization (WHO) Interim case reporting form for 2019 Novel Coronavirus (2019-nCoV) of confirmed and probable cases. https://www.who.int/docs/default-source/coronaviruse/20200121-2019-ncov-reporting-form.pdf.

[3] Yu P., Zhu J., Zhang Z., Han Y. (2020). A familial cluster of infection associated with the 2019 novel coronavirus indicating possible person-to-person transmission during the incubation period. *The Journal of Infectious Diseases*.

[4] World Health Organization Novel coronavirus (2019-nCoV): situation report, 52. https://www.who.int/docs/default-source/coronaviruse/situation-reports/20200312-sitrep-52-covid-19.pdf?sfvrsn=e2bfc9c0_4.

[5] Arai H., Ouchi Y., Yokode M. (2012). Toward the realization of a better aged society: messages from gerontology and geriatrics. *Geriatrics & Gerontology International*.

[6] Purssell E., Gould D., Chudleigh J. (2020). Impact of isolation on hospitalised patients who are infectious: systematic review with meta-analysis. *BMJ Open*.

[7] Pišot R., Marusic U., Biolo G. (2016). Greater loss in muscle mass and function but smaller metabolic alterations in older compared with younger men following 2 wk of bed rest and recovery. *Journal of Applied Physiology*.

[8] Guthold R., Stevens G. A., Riley L. M., Bull F. C. (2018). Worldwide trends in insufficient physical activity from 2001 to 2016: a pooled analysis of 358 population-based surveys with 1·9 million participants. *The Lancet Global Health*.

[9] Omura T., Araki A., Shigemoto K., Toba K. (2020). Geriatric practice during and after theCOVID‐19 pandemic. *Geriatrics & Gerontology International*.

[10] Tieland M., Trouwborst I., Clark B. C. (2018). Skeletal muscle performance and ageing. *Journal of Cachexia, Sarcopenia and Muscle*.

[11] Arango-Lopera V. E., Arroyo P., Gutiérrez-Robledo L. M., Pérez-Zepeda M. U., Cesari M. (2013). Mortality as an adverse outcome of sarcopenia. *The Journal of Nutrition, Health & Aging*.

[12] Abdelbasset W. K., Alsubaie S. F., Tantawy S. A., Elyazed T. I. A., Elshehawy A. A. (2019). A cross-sectional study on the correlation between physical activity levels and health-related quality of life in community-dwelling middle-aged and older adults. *Medicine*.

[13] McPhee J. S., French D. P., Jackson D., Nazroo J., Pendleton N., Degens H. (2016). Physical activity in older age: perspectives for healthy ageing and frailty. *Biogerontology*.

[14] Li G., Fan Y., Lai Y. (2020). Coronavirus infections and immune responses. *Journal of Medical Virology*.

[15] Mubarak A., Alturaiki W., Hemida M. G. (2019). Middle east respiratory syndrome coronavirus (MERS-CoV): infection, immunological response, and vaccine development. *Journal of Immunology Research*.

[16] Simpson R. J., Lowder T. W., Spielmann G., Bigley A. B., LaVoy E. C., Kunz H. (2012). Exercise and the aging immune system. *Ageing Research Reviews*.

[17] Saurwein-Teissl M., Lung T. L., Marx F. (2002). Lack of antibody production following immunization in old age: association with CD8^+^CD28^−^ T cell clonal expansions and an imbalance in the production of Th1 and Th2 cytokines. *Journal of Immunology*.

[18] Gayoso I., Sanchez-Correa B., Campos C. (2011). Immunosenescence of human natural killer cells. *Journal of Innate Immunity*.

[19] Jylha M., Paavilainen P., Lehtimaki T. (2007). Interleukin-1 receptor antagonist, interleukin-6, and C-reactive protein as predictors of mortality in nonagenarians: the Vitality 90+ Study. *The Journals of Gerontology Series A: Biological Sciences and Medical Sciences*.

[20] Kodama S., Saito K., Tanaka S. (2009). Cardiorespiratory fitness as a quantitative predictor of all-cause mortality and cardiovascular events in healthy men and women: a meta-analysis. *JAMA*.

[21] Pawelec G. (2006). Immunity and ageing in man. *Experimental Gerontology*.

[22] Simpson R. J., Guy K. (2010). Coupling aging immunity with a sedentary lifestyle: has the damage already been done?—a mini-review. *Gerontology*.

[23] Simpson R. J., Kunz H., Agha N., Graff R. (2015). Exercise and the regulation of immune functions. *Progress in Molecular Biology and Translational Science*.

[24] Simpson R. J., Cosgrove C., Ingram L. A. (2008). Senescent T-lymphocytes are mobilised into the peripheral blood compartment in young and older humans after exhaustive exercise. *Brain, Behavior, and Immunity*.

[25] Simpson R. J., Cosgrove C., Chee M. M. (2010). Senescent phenotypes and telomere lengths of peripheral blood T-cells mobilized by acute exercise in humans. *Exercise Immunology Review*.

[26] Spielmann G., McFarlin B. K., O’Connor D. P., Smith P. J. W., Pircher H., Simpson R. J. (2011). Aerobic fitness is associated with lower proportions of senescent blood T-cells in man. *Brain, Behavior, and Immunity*.

[27] Ludlow A. T., Zimmerman J. B., Witkowski S., Hearn J. W., Hatfield B. D., Roth S. M. (2008). Relationship between physical activity level, telomere length, and telomerase activity. *Medicine and Science in Sports and Exercise*.

[28] Haaland D. A., Sabljic T. F., Baribeau D. A., Mukovozov I. M., Hart L. E. (2008). Is regular exercise a friend or foe of the aging immune system? A systematic review. *Clinical Journal of Sport Medicine*.

[29] Shimizu K., Suzuki N., Imai T. (2011). Monocyte and T-cell responses to exercise training in elderly subjects. *Journal of Strength and Conditioning Research*.

[30] Woods J. A., Keylock K. T., Lowder T. (2009). Cardiovascular exercise training extends influenza vaccine seroprotection in sedentary older adults: the immune function intervention trial. *Journal of the American Geriatrics Society*.

[31] Shin Y. A., Lee J. H., Song W., Jun T. W. (2008). Exercise training improves the antioxidant enzyme activity with no changes of telomere length. *Mechanisms of Ageing and Development*.

[32] European Centre for Disease Prevention and Control (ECDC) Situation update worldwide. https://www.ecdc.europa.eu/en/geographical-distribution-2019-ncov-cases.

[33] Martin S. A., Pence B. D., Woods J. A. (2009). Exercise and respiratory tract viral infections. *Exercise and Sport Sciences Reviews*.

[34] Jiménez-Pavón D., Carbonell-Baeza A., Lavie C. J. (2020). Physical exercise as therapy to fight against the mental and physical consequences of COVID-19 quarantine: special focus in older people. *Progress in Cardiovascular Diseases*.

[35] Fallon K. (2020). Exercise in the time of COVID-19. *Australian Journal of General Practice*.

[36] Chen P., Mao L., Nassis G. P., Harmer P., Ainsworth B. E., Li F. (2020). Coronavirus disease (COVID-19): the need to maintain regular physical activity while taking precautions. *Journal of Sport and Health Science*.

[37] Cruz-Jentoft A. J., Baeyens J. P., Bauer J. M. (2010). Sarcopenia: European consensus on definition and diagnosis: report of the European Working Group on Sarcopenia in Older People. *Age and Ageing*.

[38] Reid K. F., Naumova E. N., Carabello R. J., Phillips E. M., Fielding R. A. (2008). Lower extremity muscle mass predicts functional performance in mobility-limited elders. *The Journal of Nutrition, Health & Aging*.

[39] Warburton D. E., Nicol C. W., Bredin S. S. (2006). Health benefits of physical activity: the evidence. *Canadian Medical Association Journal*.

[40] Pedersen B. K., Saltin B. (2015). Exercise as medicine - evidence for prescribing exercise as therapy in 26 different chronic diseases. *Scandinavian Journal of Medicine & Science in Sports*.

[41] Steeves J. A., Shiroma E. J., Conger S. A., Van Domelen D., Harris T. B. (2019). Physical activity patterns and multimorbidity burden of older adults with different levels of functional status: NHANES 2003-2006. *Disability and Health Journal*.

[42] Lee P. G., Jackson E. A., Richardson C. R. (2017). Exercise prescriptions in older adults. *American Family Physician*.

[43] Distefano G., Goodpaster B. H. (2018). Effects of exercise and aging on skeletal muscle. *Cold Spring Harbor Perspectives in Medicine*.

[44] de Vries N. M., van Ravensberg C. D., Hobbelen J. S. M., Olde Rikkert M. G. M., Staal J. B., Nijhuis-van der Sanden M. W. G. (2012). Effects of physical exercise therapy on mobility, physical functioning, physical activity and quality of life in community-dwelling older adults with impaired mobility, physical disability and/or multi-morbidity: a meta-analysis. *Ageing Research Reviews*.

[45] Vlietstra L., Hendrickx W., Waters D. L. (2018). Exercise interventions in healthy older adults with sarcopenia: a systematic review and meta-analysis. *Australasian Journal on Ageing*.

[46] Maugeri G., Castrogiovanni P., Battaglia G. (2020). The impact of physical activity on psychological health during Covid-19 pandemic in Italy. *Heliyon*.

[47] Ravalli S., Musumeci G. (2020). Coronavirus outbreak in Italy: physiological benefits of home-based exercise during pandemic. *Journal of Functional Morphology and Kinesiology*.

[48] Lesser I. A., Nienhuis C. P. (2020). The impact of COVID-19 on physical activity behavior and well-being of Canadians. *International Journal of Environmental Research and Public Health*.

[49] Giustino V., Parroco A. M., Gennaro A., Musumeci G., Palma A., Battaglia G. (2020). Physical activity levels and related energy expenditure during COVID-19 quarantine among the Sicilian active population: a cross-sectional online survey study. *Sustainability*.

[50] Mujika I., Padilla S. (2000). Detraining: loss of training-induced physiological and performance adaptations. Part II. *Sports Medicine*.

[51] Paoli A., Musumeci G. (2020). Elite athletes and COVID-19 lockdown: future health concerns for an entire sector. *Journal of Functional Morphology and Kinesiology*.

